# The hidden toll of air pollution: mental health effects on middle-aged and older adults

**DOI:** 10.3389/fpubh.2025.1610330

**Published:** 2025-07-04

**Authors:** Xuan Zou, Yao He, Haoyang Lu

**Affiliations:** ^1^School of Economics and Trade, Hunan University, Changsha, Hunan, China; ^2^Department of Economics, University of Bath, Bath, United Kingdom

**Keywords:** air pollution, mental health, older adults, CHARLS, vulnerable groups

## Abstract

**Objective:**

Depressive disorders are increasingly recognized as a major public health challenge, especially in aging societies. This study aims to explore the impact of air pollution on the mental health of middle-aged and older adults, with a particular focus on identifying the vulnerable subgroups and underlying mechanisms.

**Methods:**

We employ micro-level data to empirically examine how exposure to fine particulate matter (PM2.5) affects short-term mental health outcomes, as measured by the Centers for Epidemiologic Studies Depression Scale (CES-D). We also explore the potential mediating channels through which air pollution may influence psychological well-being, including sleep quality, life satisfaction, physical health, and cognitive functioning.

**Results:**

Our findings show that the increment in fine particulate matter (PM2.5) increases the scores of Centers for Epidemiologic Studies Depression Scale (CES-D), indicating a deterioration in mental health. The negative effects of air pollution are particularly pronounced among older adults, females, and widowed individuals. Mechanism analysis reveals that air pollution significantly worsens sleep quality, reduces life satisfaction, impairs cardiopulmonary health, and diminishes cognitive competence.

**Conclusions:**

This study highlights the substantial mental health burden of air pollution, especially for vulnerable populations. We find suggestive evidence that improving access to pollution information and urban greening can help mitigate these adverse effects. Our findings underscore the urgent need for targeted public health interventions and environmental policies to address the mental health consequences of air pollution, particularly in the context of climate change and population aging.

## 1 Introduction

A large body of literature documents that mental disorders have become the major global burden of disease (1). The adverse effects of depressive disorders are likely to have a substantial impact on economic activities. Untreated mental disorders can lead to profound unhappiness, a marked decrease in the enjoyment of life, and the deterioration of essential social bonds (2–4). It can further give rise to troubles in the socio-economic domain, resulting in absenteeism, reduced productivity, or even job loss (5–7). Moreover, middle-aged and older adults are more likely to suffer from a wide range of mental and psychosocial adjustment disorders, and only about 10% of them seek medical attention (8, 9). At the same time, ambient air pollution is considered a global public health issue in developed and developing countries (10). Air pollution can impair human physical condition, including respiratory illness, stroke, pulmonary disease, and so on (11–13). However, the mitigating behaviors that can reduce the negative impact of air pollution on mental illness are not yet clear. Exploring the mitigating behaviors of the negative impact of air pollution on mental illness is unclear. Addressing this gap is crucial to comprehending how air pollution influences mental states, particularly for middle-aged and older adults, who represent a significant portion of the population.

This study provides empirical evidence on how air pollution affects short-run mental health outcomes among the middle-aged and older adults. We examine how air pollution has an impact on mental health by employing panel data at the individual level from the China Health and Retirement Longitudinal Study (CHARLS) and corresponding city-level air pollution statistical data. Specifically, we construct a ten-item version of the Center for Epidemiologic Studies Depression Scale (CES-D), which serves as a composite indicator reflecting the level of mental health. The higher scores of CES-D indicate more severe depressive symptoms. Meanwhile, the PM2.5 concentration and air quality index (AQI) data are used as a proxy for air pollution. AQI data are derived from real-time monitoring data published on the official website of the China National Environmental Monitoring Center.^1^ The PM2.5 concentration data are estimated using satellite information, ensuring that the measurements are accurate and not subject to human manipulation (14). Our methodology relies on two sources of data processing. First, we assign a monthly average PM2.5 concentration to each participant using the interview data and participants' locations provided by CHARLS. Second, in practice, the dissemination of air pollution exhibits evident spatial spillover effects, and meteorological conditions significantly influence the dispersion and transportation of air pollutants. Therefore, we control for all meteorological variables that may affect the dispersion and transportation of air pollutants, including sunshine hours, relative humidity, wind speed, and precipitation. This ensures that our empirical results are accurate through rigorous data processing.

This study makes contributions on three fronts. First, we relate to the literature between air pollution and mental illness. Many researchers have explored the direct negative impact of air pollution (15). Different air contaminants, including fine airborne particulate matter (PM2.5, PM5, and PM10), sulfur dioxide, carbon monoxide (CO), benzene, and ozone, have been shown to be toxic to the central nervous system (CNS) (16, 17). Long-term exposure to air pollution has adverse effects on health, including an increased risk of lung cancer (18). Of note, the detrimental impact of air pollution on individual performance and economic outcomes has been documented (19, 20). In particular, air pollution reduces reading and math outcomes for schoolchildren due to lower school attendance and inattentiveness (21), and it further undermines human capital as a potential consequence of poor performance in innovation activities (22). Emerging research has tried to investigate the association between air pollution and health outcomes (23). Air pollutants are negatively associated with cognitive development (24). Also, the association between exposure to air pollution and anxiety has been suggested (25). However, the results are inconsistent (26, 27). Our paper highlights the importance of emphasizing heterogeneous effects in different subgroups when measuring the mental health consequences of air pollution using a large-scale longitudinal dataset. This allows us to provide novel insights into how air pollution exposure can affect the mental health status of older people.

Second, this study examines the factors that influence mental health outcomes. Previous studies have investigated factors such as sex (28), education (29), ethnicity (30), smoking and drinking frequency (31), and social participation (32). Additionally, environmental factors such as living conditions (town vs. village living) (33), crime rate (34), unemployment rate (35), and household income inequality (36) significantly influence mental health over time. We provide empirical estimations to explore the underlying mechanisms through which air pollution affects mental illness from the individual perspective. We point out that poor sleep quality, decreased life satisfaction, reduced physical fitness, and declining cognitive ability are major drivers of deteriorating mental health among the middle-aged and older adults. Furthermore, we distinguish the differences in impacts on the middle-aged and older adults and propose that late-life depression should be a senior issue that the public and researchers should pay more attention to.

Third, it is unrealistic to eliminate air pollution thoroughly, as human activities and natural processes contribute to some extent to pollutant emissions. Based on these considerations, our study provides empirical evidence on how people might respond to air pollution exposure, focusing on two mitigation measures: private access to pollution information and the filtering effect of urban forests. These responses are still largely unknown to researchers. We first document the private access to pollution information to increase people's awareness about pollution issues. These changes trigger people's avoidance behaviors in response to air pollution, alleviating psychological problems. We propose another human intervention to mitigate pollution exposure: urban forests, which are among the most prevalent urban afforestation policies. Urban forests will absorb hazardous chemical pollutants and effectively cut off diffusion pathways for particulate pollution. The findings could help design more effective public health campaigns and policies addressing environmental and mental health challenges.

The remainder of the paper is structured as follows: Section 2 provides an overview of the background. Section 3 outlines the data. Section 4 details the empirical strategy and baseline findings. Section 5 explores the robustness checks. Section 6 examines the heterogeneity analyses. Section 7 delves into the potential mechanisms. Section 8 demonstrates further analyses. Section 9 concludes the paper.

## 2 Background

### 2.1 Air pollution

**Figure 1:**
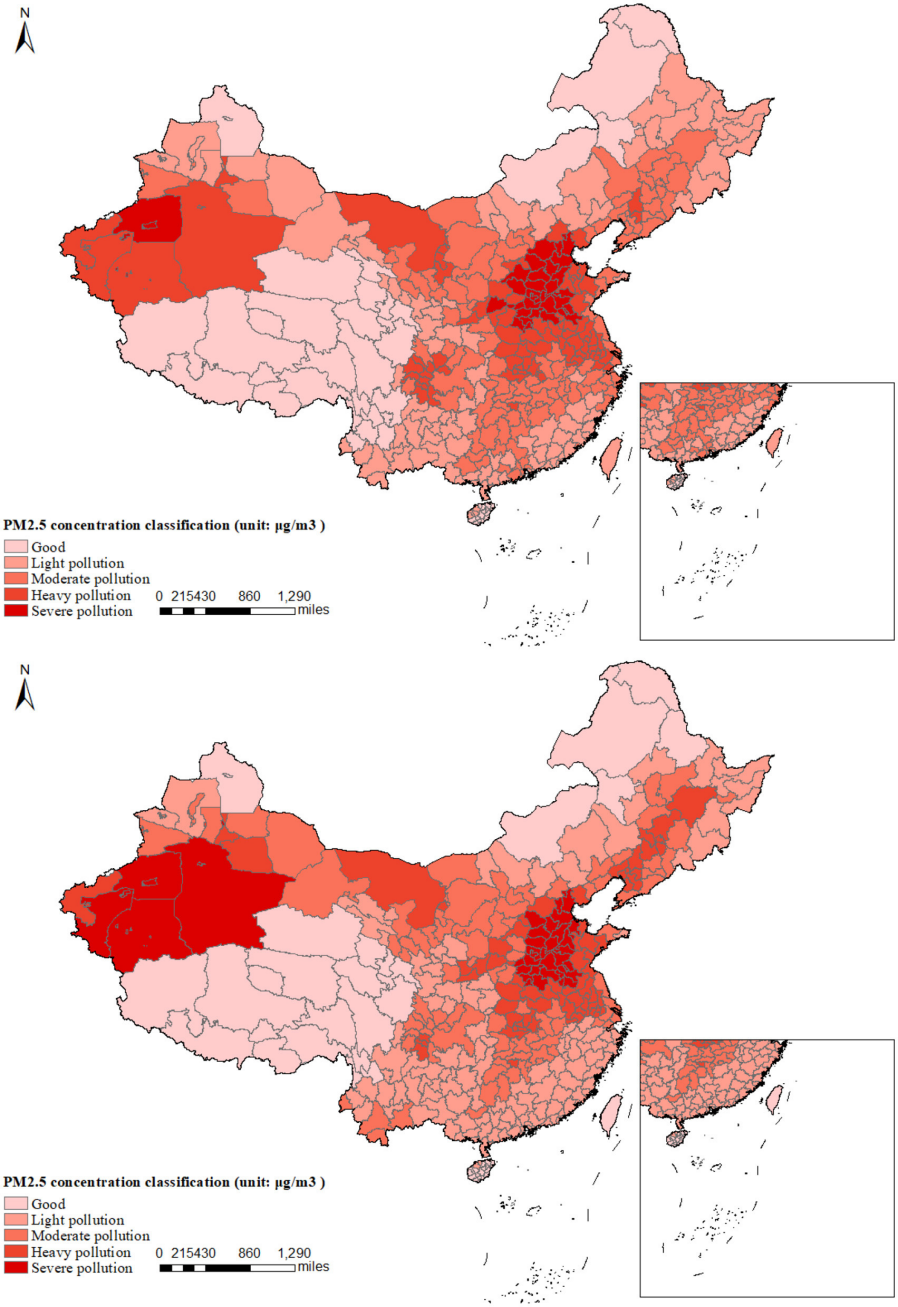
Comparison of average PM2.5 concentrations: upper panel (2000) vs. lower panel (2020). This figure presents a comparative analysis of the annual average PM2.5 concentrations across prefecture-level cities in China for the years 2000 and 2020. The comparison indicates that there has been an improvement in PM2.5 concentrations in the northern and eastern regions of China in 2020 compared to the year 2000. Conversely, the western regions have experienced an expansion in areas of severe pollution over the past two decades.

**Figure 2:**
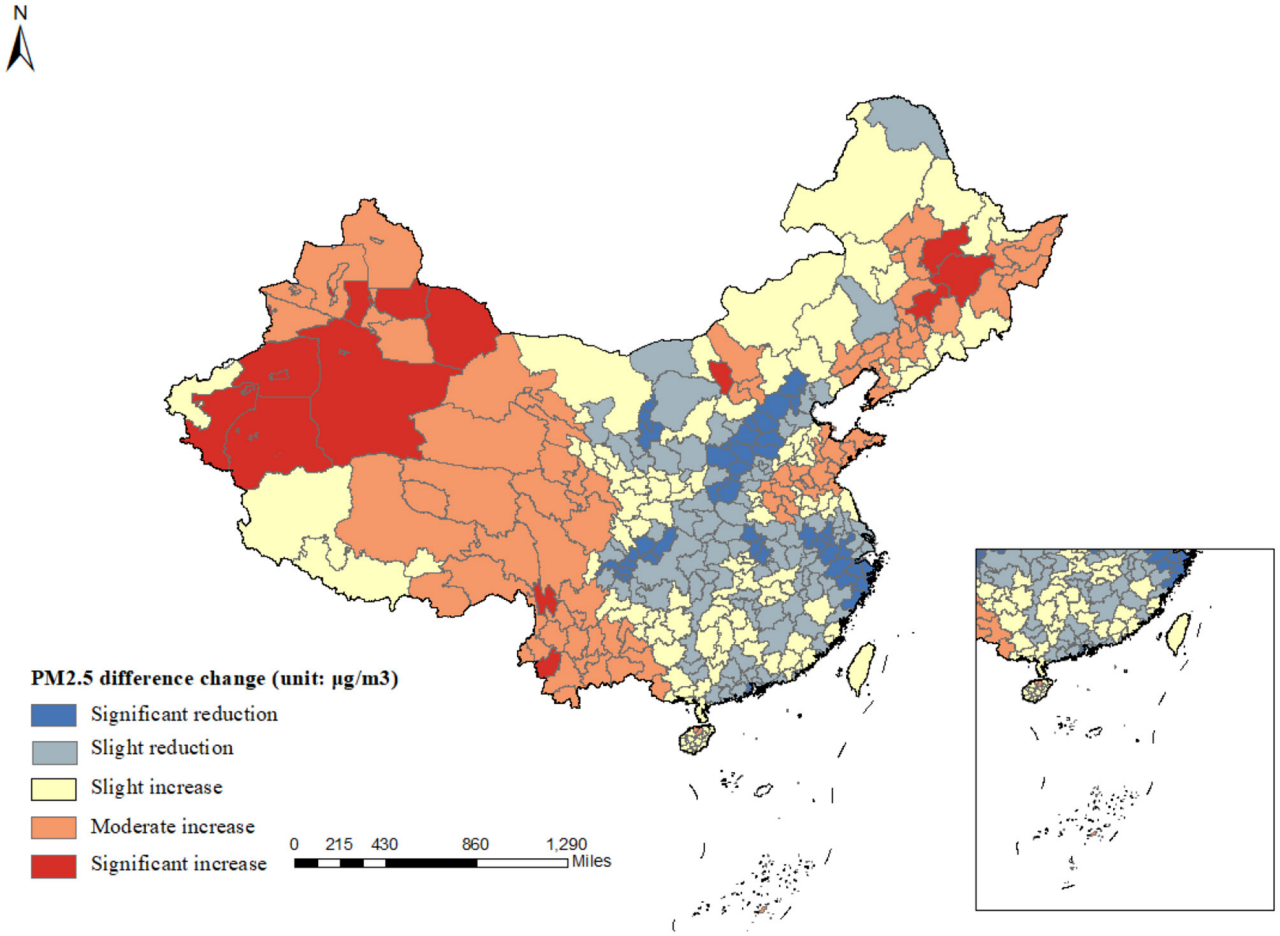
Average PM2.5 difference change in 2000 and 2020. This figure displays the spatial distribution of PM2.5 concentration changes in China from 2000 to 2020. Pollution has intensified in the western region, whereas the central and eastern areas have shown initial success in pollution control efforts, with a reduction in PM2.5 concentrations. However, regions of North China continue to experience severe pollution.

During the past decades, China has experienced unprecedented economic growth, but it has also brought with it the byproduct of severe air pollution (37). PM2.5, as the main component of air pollution, has caused substantial welfare losses to individuals and society (38). The comparison of PM2.5 concentration maps for China in 2000 and 2020 reveals a substantial improvement in air quality over the two decades in Figure 1. The 2020 map shows a significant reduction in severe and heavy pollution areas, especially along the southeastern coast. This trend indicates the effectiveness of environmental policies and measures in reducing pollution, although some regions still require further attention to achieve better air quality standards. Furthermore, Figure 2 illustrates the difference in the annual average PM2.5 concentration level across cities of China in 2000 and 2020. In general, air pollution concentrations have significantly increased over the period. Moreover, there is a striking spatial disparity in the PM2.5 level.

To address this problem, China has implemented several policies to solve the issue of air pollution. For example, the Two Control Zone (TCZ) policy was launched in 1998, designating certain geographic areas where strict measures are applied to control pollutants, including PM2.5, sulfur dioxide (SO_2_), and nitrogen oxides (NOx). China has continuously implemented various air quality action plans, including the National Air Pollution Control Action Plan (2013–2017), which set targets for reducing PM2.5 levels in critical regions, such as Beijing-Tianjin-Hebei, the Yangtze River Delta, and the Pearl River Delta. China has implemented stricter vehicle emission standards and promoted the use of electric vehicles (EVs) to address its significant air quality challenges.

### 2.2 Aging problem

**Figure 3:**
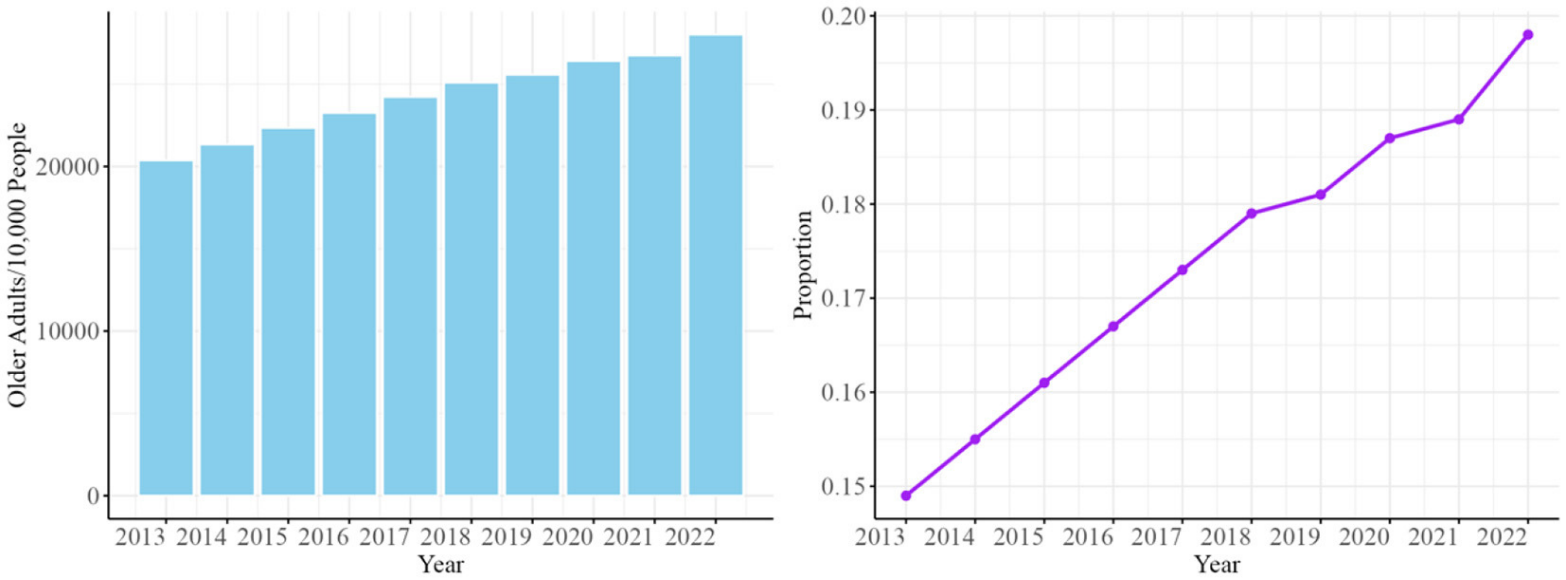
The proportion of China's older adults population from 2013 to 2022. Data Source: National Bureau of Statistics of China. Data Source: National Bureau of Statistics of China. This figure presents the interannual changes in the total number of older adults **(left panel)** and their proportion within the total population **(right panel)** in China from 2013 to 2022. It reflects the ongoing acceleration of the aging population in China. The left panel illustrates the growth in the absolute number of older adults. The right panel demonstrates the increasing proportion of older adults within the overall population.

China is experiencing significant demographic aging, with its population structure undergoing a dramatic transformation. Figure 3 displays the proportion of China's older adults population to the total population from 2013 to 2022. There are an increasing number of older adults. The working-age population is expected to decline by 23% from 925 million in 2011 to 800 million in 2050 (39). This demographic shift is primarily driven by two factors. First, the one-child policy, implemented from 1979 to 2015 to control population growth, significantly reduced birth rates. Second, advancements in healthcare have improved life expectancy, contributing to the rise in the older adults. Additionally, declining mortality rates further accelerate this trend. According to the Seventh National Population Census published in 2021, China's total fertility rate (TFR) dropped from 5.6 in 1970 to an estimated 1.3 in recent years. This demographic transition presents both challenges and opportunities. It calls for comprehensive reforms in social care, healthcare systems, and economic strategies to support the aging population. At the same time, advancements in technology and healthcare offer opportunities to enhance the quality of life for older adults.

### 2.3 Mental health status of the middle-aged and older adults

The prevalence of depression among the middle-aged and older adults in China is a significant concern. The pooled overall prevalence of depressive symptoms among older adults in China was ~20.0%, indicating a considerable burden of depression in this demographic (40). The incidence of depressive symptoms has shown a slow increase since the 1990s. Especially, the evolution of depression among the Chinese older adults from 2011 to 2018 revealed a significant upward trend in the detection rates of depression, reaching 44.5% by 2018 (41). The prevalence of depression in the middle-aged and older adults population is of even greater concern. There is a clear need for policies and programs that consider the specific needs of vulnerable older adults, focusing on improving social participation and access to mental health services to mitigate the risk of depression. We provide new perspectives to explore how air pollution, as a potential causative factor, affects the mental health status of the middle-aged and older adults.

## 3 Data analysis

**Figure 4:**
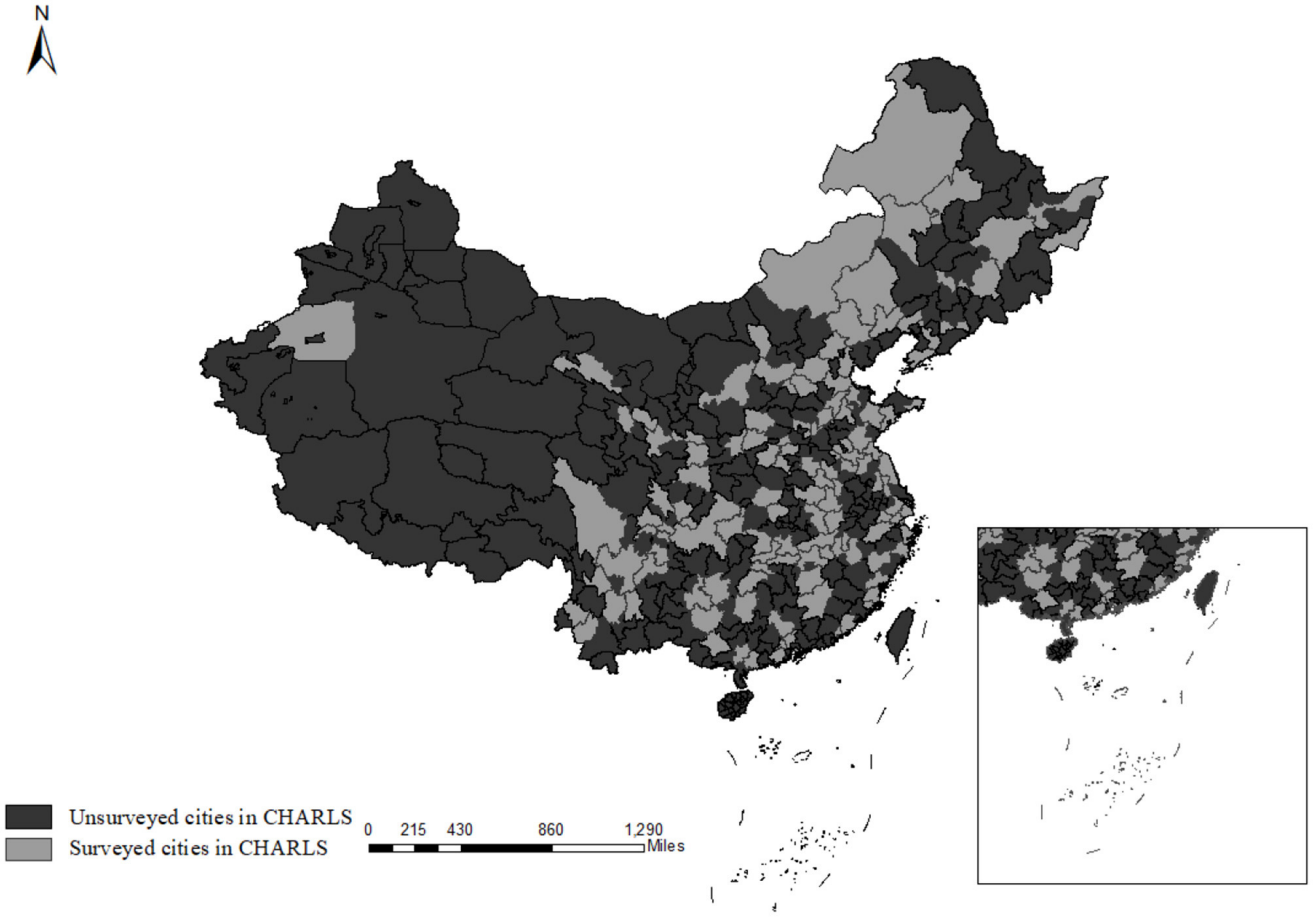
Distribution of Chinese cities surveyed by CHARLS. This figure illustrates the spatial distribution of cities covered by the China Health and Retirement Longitudinal Study (CHARLS). Light gray areas denote cities that CHARLS has surveyed, while dark gray areas represent cities that have not been included in the survey. CHARLS encompasses 28 provinces, indicating a broad coverage across China.

Data on mental health and individual characteristics of the middle-aged and older adults are obtained from CHARLS. Initiated in 2011, CHARLS, a nationally bi-annual longitudinal survey, collects a representative sample of persons in China 45 years of age or older and their spouses, including a series of assessments of social, economic, and health conditions. The CHARLS involves about 10,000 households and 17,500 individuals in 150 counties (districts) and 450 villages (resident committees). Overall, CHARLS is a nationally comprehensive longitudinal social survey that is intended to provide a high-quality public micro-database to meet the needs of scientific and policy research about aging-related issues (42). Figure 4 depicts the cities in China that were involved in CHARLS.

We use survey data from the 2013, 2015, 2018, and 2020 waves of CHARLS. To maintain sample coherence, we restrict our sample to individuals who participated in all four survey waves. These waves consistently feature the same ten questions that enable the construction of the CES-D (Center for Epidemiologic Studies Depression) scale, which is also recognized as the K10 instrument—a 10-question screening scale for psychological distress (43). Drawing on the findings of Chen and Mui (44), we argue that the CES-D 10 scale is a reliable and valid measure of mental health status among middle-aged and older adults in China. The ten questions in CHARLS applied to measure the mental health status are as follows: in the last 30 days, how often did you feel (a) bothered by things, (b) trouble keeping mind, (c) depressed, (d) effortless, (e) that everything I did was an effort, (f) hopeless about the future, (g) fearful, (h) restless, (i) unhappy, (j) lonely and that I cannot go on with my life. Each question takes the value of 1 if the participant matched such a feature for each question most or all the time. Each question takes the value of 0.5 if the participant occasionally or a little of the time matched such a characterization for the question. If the participant is rare or does not have such a characteristic, the question takes the value 0. Based on the participants' answers, we construct the CES-D score as the sum of the answers to the ten questions. A higher score CES-D score indicates a higher risk of mental health.

The core explanatory variable is the air pollution level. It is measured by the monthly average surface concentration of PM2.5. Specifically, the PM2.5 concentrations in each city were extracted according to the vector map of urban administrative regions in China, which comes from the 0.01^°*^ 0.01° grid data of ground-level PM2.5 obtained from the University of Washington ACAG website.^2^ We adopt the satellite monitoring data, which can more accurately reflect the PM2.5 concentration level and urban trend and solve the problem of the lack of historical data on PM2.5 concentration.

The demographic characteristics include the hukou type (rural or urban), whether individuals receive (or expect to receive) or contribute to the pension^3^, and medical insurance^4^. Precisely, whether to receive a pension reflects an individual's post-retirement financial stability and income security. Individuals with adequate pension income may also experience a greater sense of autonomy, life satisfaction, and reduced psychological distress. Whether to receive medicare variables represents access to health insurance coverage for individuals. It is a key proxy for healthcare access, influencing the affordability and utilization of medical services. Coverage can reduce out-of-pocket expenses and make treatments such as counseling, psychiatric care, or medication more accessible.

The monthly urban meteorological characteristics are attained from the China Meteorological Data Sharing Service System (CMDSSS). They include (a) average sunshine hours (sunshine duration): longer sunshine hours result in less weakening of solar radiation, inhibiting ozone generation and improving air quality. (b) Average relative humidity (Avg. humidity): the lower relative humidity can promote the settlement and diffusion of inhalable particles in the air, thus improving air quality. (c) Wind speed (Avg. wind speed): wind speeds create air movement and cause pollutants to spread. (d) Average precipitation (precipitation): sufficient precipitation leads to lower surface air temperatures and the obvious effect of wet deposition, which is conducive to reducing the concentration of particulate matter in the air. Furthermore, we performed a multicollinearity test on the weather-related variables, with all VIFs falling below 3, indicating that the intercorrelations among these variables are sufficiently low to not introduce substantial uncertainty into our empirical strategy.

We compile multiple data sets to allow for a comprehensive study of the impact of air pollution on mental health among middle-aged and older adult groups. Table 1 presents the summary statistics of the data used in our baseline empirical regression.

**Table 1:** Summary statistics.

**Variable**	**Obs**	**Mean**	**SD**	**Min**	**Max**
**Panel A: mental health variable**					
CES-D	66,504	2.9186	1.5137	0	10
Bothered	66,504	0.2985	0.3536	0	1
Distracted	65,187	0.2904	0.3514	0	1
Depressed	65,687	0.2876	0.3437	0	1
Effortless	65,771	0.2962	0.3694	0	1
Hopeless	64,045	0.4704	0.4185	0	1
Fearful	66,236	0.1174	0.2624	0	1
Restless	66,292	0.3542	0.3906	0	1
Unhappy	65,952	0.6114	0.3942	0	1
Lonely	65,944	0.1779	0.3156	0	1
Stagnant	65,624	0.1242	0.2734	0	1
**Panel B: air pollution variables**					
PM2.5	66,504	22.243	39.603	1.394	717.869
**Panel C: control variables**					
Sunshine duration	64,437	192.9585	49.9982	27.9342	359.9217
Precipitation	64,437	176.3738	106.0827	0.2090	745.7543
Avg. humidity	64,437	77.0348	7.1659	41.6927	92.8855
Avg. wind speed	64,437	4.8029	1.0028	2.6610	8.6429
Hukou	48,396	1.9360	3.2312	1	17
Pension	66,466	0.4719	0.4992	0	1
Medicare	66,504	0.9250	0.2634	0	1

## 4 Methods

### 4.1 Empirical strategy

Our empirical objective is to examine the mental health effects of air pollution. It is typically measured using “pollution gradients,” i.e., $\frac{\partial MentalHealth}{\partial Pollution}$ for pollution's mental health damage (45). We focus on this marginal approach and estimate how air pollution on mental health.

Taking advantage of the regional and temporal variations in air quality, this paper seeks to identify the impacts of air pollution on the mental health of middle-aged and older adults. Our empirical strategy anchors on a fixed-effect model. The model is given as follows:


(1) $$\begin{array}{l} MH_{icym}=\beta_{0}+\beta_{1}pollution_{cym}+\beta_{2}X_{icmy}+\beta_{3}W_{cmy}+\delta_{c}T_{t}+\mu_{i}\\ \;+\sigma_{c}+\pi_{y}+\omega_{m}{+\varepsilon }_{icmy} \end{array}$$

In this specification, the outcome variable *MH*_*icym*_ denotes the mental health of middle-aged and older adults i from city *c* in month m year *y*. *pollution*_*cym*_ represents the monthly mean fine particulate matter (PM2.5). The coefficient, β_1_, captures the depression effect of air pollution. The vector *X*_*icmy*_ refers to individual controls. The vector *W*_*cmy*_ refers to weather characteristics, including the average humidity, sunshine hours, wind speed, and precipitation. We further control for individual fixed effects (μ_*i*_) to account for unobservable factors that are constant over time at the individual level, city fixed effects (σ_*c*_) to account for unobservable factors that are constant over time at the city level, and year (π_*y*_) and month fixed effects (ω_*m*_) to control for external factors that are common to all units of observation in specific years and months. *T*_*t*_ refers to the monthly time trend that the interview date falls. ε_*icmy*_ is the error term. We cluster standard errors at the city level.

### 4.2 Baseline results

First, we investigate the baseline results of how air pollution affects the mental health of middle-aged and older adults. Table 2 presents the corresponding estimated coefficients for CES-D scores. Column (1) presents a parsimonious model that controls for the PM2.5 variables, individual FE, city FE, month FE, and year FE. Column (2) additionally controls the meteorological characteristics. Column (3) adds individual characteristics. Column (4) further controls for the linear monthly time trends. The increase in PM2.5 concentration leads to the CES-D score rising by 0.0168, 0.0176, 0.0313, and 0.0313 in columns (1) to (4), respectively. In conclusion, the model with tighter controls leads to a higher CES-D score. This implies that PM2.5 increases CES-D scores, which is statistically significant. From a mental health perspective, the magnitude of this effect is substantial. For example, according to the findings of Zhang et al. (46), the proportion of China's population living in areas with high PM2.5 concentrations (annual average PM2.5 concentration exceeding 75 μg/m3, compared to the WHO-recommended annual average of ≤ 5 μg/m3) accounted for ~5% of the total population after 2015. This exposure level suggests that elevated PM2.5 concentrations could significantly exacerbate depressive symptoms. It further verifies that air pollution exposure poses a potential threat to the mental health of the middle-aged and older adults.

**Table 2:** Average effect of air pollution exposure on CES-D scores.

**Variables**	**(1)**	**(2)**	**(3)**	**(4)**
Pollution	0.0168^***^	0.0176^***^	0.0313^***^	0.0313^***^
	(0.0062)	(0.0058)	(0.0070)	(0.0058)
Sunshine duration		−0.00069^**^	−0.00041^**^	−0.0004
		(0.00034)	(0.0003)	(0.0004)
Precipitation		−0.000236^*^	−0.00012	−0.00011
		(0.00015)	(0.0002)	(0.00016)
Avg. humidity		0.00103	0.0012	0.0012
		(0.0028)	(0.0032)	(0.0032)
Avg. wind speed		0.01939	−0.0194	−0.0193
		(0.02833)	(0.0336)	(0.0336)
Hukou			−0.0004	−0.0004
			(0.0023)	(0.0023)
Pension			−0.0670^***^	−0.0670^***^
			(0.0231)	(0.0231)
Medicare			−0.0669^**^	−0.0669^**^
			(0.0319)	(0.0319)
Constant	2.8915^***^	2.8863^***^	2.9776^***^	2.9747^***^
	(0.1367)	(0.2886)	(0.3492)	(0.3505)
Individual FE	YES	YES	YES	YES
City FE	YES	YES	YES	YES
Year FE	YES	YES	YES	YES
Month FE	YES	YES	YES	YES
Monthly time trend	NO	NO	NO	YES
Observations	62,108	60,173	40,791	40,791
Adj. *R*^2^	0.4480	0.4458	0.4319	0.4319

Standard errors in parentheses are clustered at the city level. Robust standard errors clustered at the city level. Individual FE, City FE, Year FE, and Month FE denote the inclusion of individual fixed effects, city fixed effects, annual fixed effects, and monthly fixed effects, respectively. YES, indicates that the corresponding fixed effect is controlled for, while NO indicates that it is not controlled for. ^***^P < 0.01; ^**^P < 0.05; ^*^P < 0.1.

## 5 Robustness checks

To test the stability of the baseline estimates, we perform a series of robustness checks, including distinguishing air pollution levels, alternative measures, alternative model specifications, and falsification tests.

### 5.1 Distinguishing symptoms of depression

The major components of depression include depressed mood, feelings of guilt and hopelessness, feelings of meaninglessness, and loneliness, which were identified from the clinical literature and factor analytic studies (47). The K-10 questions contain an inquiry about the frequency of these depressive symptoms. We next explore the effects of air pollution on the degree of each depression symptom separately. Table 3 displays the results, where the larger coefficients indicate more severe symptoms of depression. We find that air pollution significantly increases the occurrences of depressive symptoms of bothered, lonely, depressed, and meaningless.

**Table 3:** The effect of air pollution on specific depressive symptoms.

**Variables**	**Bothered**	**Lonely**	**Depressed**	**Hopeless**	**Meaningless**
Pollution	0.0196^**^	0.0155^**^	0.0204^***^	0.0109	0.0181^***^
	(0.0060)	(0.0064)	(0.0040)	(0.0069)	(0.0060)
Constant	1.970^***^	1.268^***^	1.916^***^	2.337^***^	2.162^***^
	(0.2335)	(0.2091)	(0.1963)	(0.3547)	(0.2777)
Controls	YES	YES	YES	YES	YES
Individual FE	YES	YES	YES	YES	YES
City FE	YES	YES	YES	YES	YES
Year FE	YES	YES	YES	YES	YES
Month FE	YES	YES	YES	YES	YES
Time trend	YES	YES	YES	YES	YES
Observations	40,791	40,370	40,106	38,873	40,272
Adj. *R*^2^	0.3000	0.3159	0.2983	0.2433	0.3071

The controls of all specifications include individual and meteorological characteristics. The individual characteristics include the hukou type, availability of pension, and health insurance. The meteorological characteristics include sunshine hours, relative humidity, wind speed, and precipitation. Robust standard errors clustered at the city level. Individual FE, City FE, Year FE, and Month FE denote the inclusion of individual fixed effects, city fixed effects, annual fixed effects, and monthly fixed effects, respectively. YES indicates that the corresponding fixed effect is controlled for, while NO indicates that it is not controlled for.

^**^P < 0.05, ^****^P < 0.01.

### 5.2 Classifying air pollution levels

We further used Air Quality Index (AQI), which was later substituted in robustness checks to strengthen the baseline results. The AQI data were obtained from the China National Environmental Monitoring Center through the web crawler technology, which can provide a broader measure of air quality, capturing multiple pollutants beyond PM2.5 alone. We try to validate the robustness of the findings, examining whether the effects identified with PM2.5 hold when evaluated against a more comprehensive air quality index. This approach enables an inclusive assessment of pollution-related impacts on mental health outcomes. Specifically, the AQI tracks the levels of various air pollutants, including ground-level ozone, particulate matter (such as PM2.5 and PM10), carbon monoxide, sulfur dioxide, and nitrogen dioxide. We categorize the AQI value into levels of increasing health concern. When the AQI value is from 0 to 50, the air quality is considered satisfactory, and air pollution poses little or no risk. The AQI value is from 51 to 100. The air quality is acceptable; however, there may be some health concerns for a very small number of people who are unusually sensitive to air pollution. The AQI value is from 101 to 150. The air quality is unhealthy for sensitive groups, but the public is unlikely to be affected. The AQI value is from 151 to 200. The air quality is unhealthy, and everyone may experience health effects. The AQI value is from 201 to 300. The air quality is very unhealthy; everyone may experience more serious health effects. The AQI value is from 301 to 500. The air quality is hazardous, which is a health warning of emergency conditions.

We use the following model to examine the effects of different pollution gradients on mental health:


(2) $$\begin{array}{l} MH_{icym}=\beta_{0}+\sum_{k}\beta_{k}{*pollution}_{cym}+\beta_{2}X_{icmy}+\beta_{3}W_{cmy}+\delta_{c}T_{t}\\ \;+\mu_{i}+\sigma_{c}+\pi_{y}+\omega_{m}{+\varepsilon }_{icmy} \end{array}$$

Since the AQI value is from 0 to 50, which is no potential threat to the public, we use the 0–50 (excellent air quality) as the baseline group. For each observation, *pollution*_*cym*_ represents the air quality falling into the kth bin of {301–500 (seriously unhealthy,); 201–300 (unhealthy); 151–200 (moderately unhealthy); 101–150 (slightly unhealthy); 51–100 (acceptable)}. The rest of this model is consistent with Equation 1. Table 4 presents the results indicating that severe air pollution levels were associated with higher CES-D scores and more pronounced depressive symptoms, worsening mental health status. Moreover, the results may suggest that the relationship between air pollution levels and mental health outcomes might not be strictly linear across different pollution bins.

**Table 4:** The impact of different air pollution levels on different mental symptoms.

**Variables**	**CES-D**	**Meaningless**	**Lonely**	**Depressed**	**Bothered**
(301–500) seriously unhealthy	1.0004^***^	0.0308	−0.0314	0.0474	1.0622^***^
	(13.9620)	(0.0510)	(−0.8988)	(0.0393)	(0.0583)
(201–300) unhealthy	0.0099	0.0089	0.0950^***^	−0.0517	−0.0074
	(0.1344)	(0.0499)	(2.8102)	(0.0437)	(0.0908)
(151–200) moderately unhealthy	−0.0159	−0.0283	0.0754^***^	−0.0561	−0.1291
	(−0.3052)	(0.0402)	(3.1528)	(0.0306)	(0.0763)
(101–150) slightly unhealthy	−0.0872	−0.0767	0.0341	−0.1160^***^	−0.1332
	(−1.5241)	(0.0433)	(1.2610)	(0.0360)	(0.0806)
(51–100) acceptable	−0.2046^**^	−0.2134^***^	−0.0060	−0.0266	−0.2121^**^
	(−2.1876)	(0.0385)	(−0.1896)	(0.0473)	(0.0689)
Observations	40,791	40,272	40,791	40,791	38,873
Adj. R^2^	0.4315	0.3071	0.3274	0.2981	0.2752

The AQI stands for Air Quality Index. The control air pollution bin is excellent (AQI ≤ 50). All specifications include demographic characteristic. The individual characteristics include the hukou type, availability of pension, and health insurance. The meteorological characteristics include sunshine hours, relative humidity, wind speed, and precipitation. All estimates include individual, city, year, and month fixed effects and linear monthly trends. Robust standard errors clustered at the city level. Individual FE, City FE, Year FE, and Month FE denote the inclusion of individual fixed effects, city fixed effects, annual fixed effects, and monthly fixed effects, respectively. YES indicates that the corresponding fixed effect is controlled for, while NO indicates that it is not controlled for.

^**^P < 0.01, ^***^P < 0.001.

### 5.3 Placebo test

**Figure 5:**
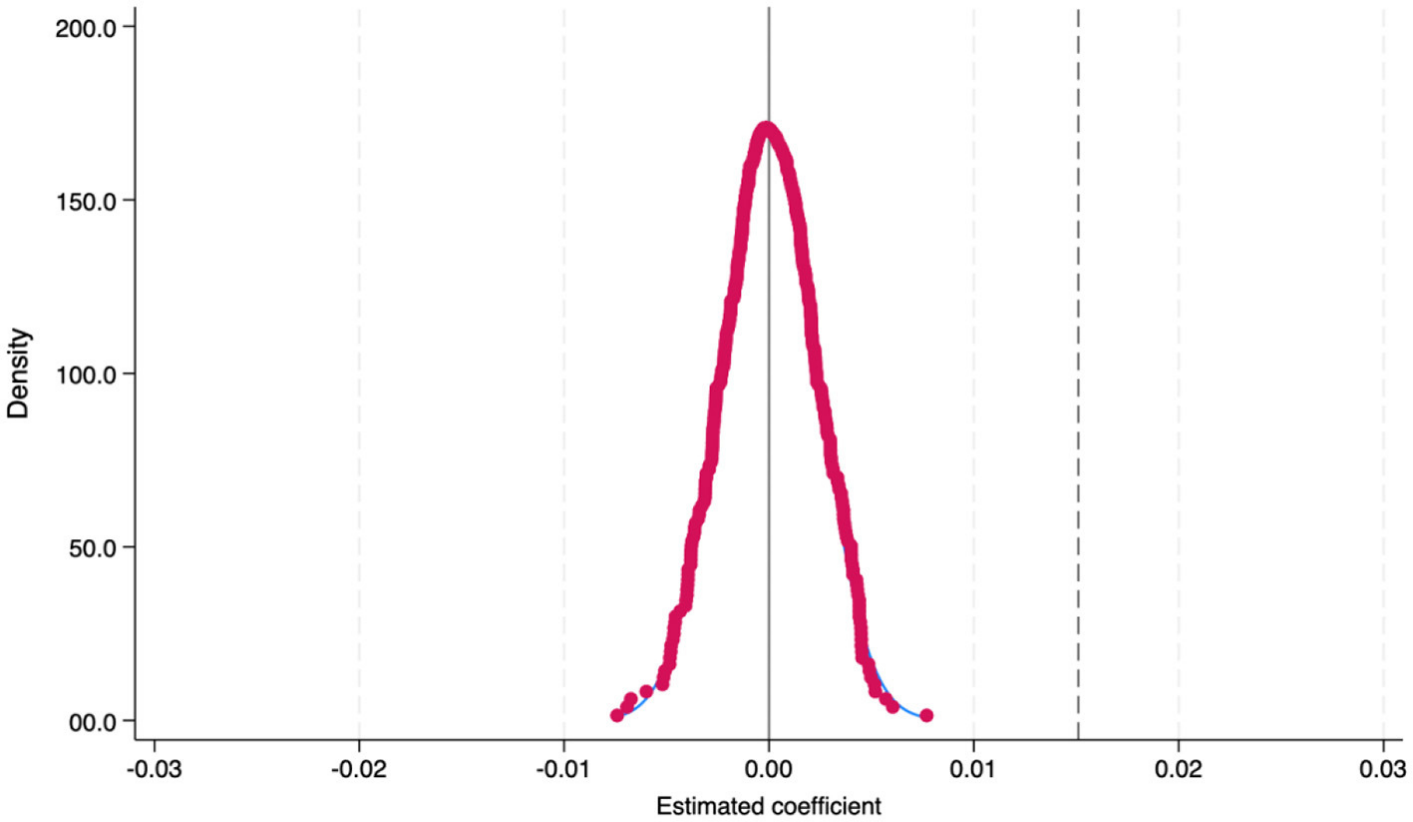
Placebo test results. This figure presents the results of the placebo test, demonstrating that the distribution of the coefficients adheres to a normal distribution centered around zero, which is significantly distant from the vertical dashed line that represents the actual coefficient estimate.

Although we have added adequate control variables at the city level that can affect air pollution, the empirical strategy may still be confounded by other unobservable factors that affect local air pollution and thus lead to estimation bias. Following Ferrara et al. (48), we use the placebo test by randomly assigning PM2.5 values. If no other factors disturb the policy, the coefficient will equal zero, meaning = 0. Next, we repeated the regression of Equation 1 500 times. In each round, the values of PM2.5 are shuffled across the city, year, and month, and the estimated coefficient is recorded. Figure 5 displays the distribution of these estimates, where the distribution of the coefficients follows a normal distribution with zero as the mean, which is far from the vertical dashed line indicating the actual coefficient estimate obtained from Column (4) in Table 2. This suggests that our identification is unbiased, and no unobserved characteristics affect our empirical results. It further verifies that the significant actual estimates rightfully refer to the impacts of air pollution on mental health rather than mere coincidence.

We consider another falsification test. Future air pollution is usually difficult and does not directly affect the current mental health status. Based on this, we follow Burnet and Kogan (49) and use the 2-year forward PM2.5 value as an explanatory variable in Equation 2 to conduct the falsification test. The results in column (1) of Table 5 signify that air pollution does not significantly affect mental health two years forward. This falsification test further demonstrates that our conclusions are not confounded by unobserved factors related to air pollution.

**Table 5:** Robustness check: falsification test and excluding unpredictable shocks.

**Variables**	**Falsification test**	**Excluding shocks**
Pollution	−0.0016	0.0157^**^
	(0.1210)	(0.0006)
Constant	3.0758^***^	2.7542^***^
	(0.3772)	(0.4893)
Controls	YES	YES
Individual FE	YES	YES
City FE	YES	YES
Year FE	YES	YES
Month FE	YES	YES
Time trend	YES	YES
Observations	40,791	21,444
Adj. R^2^	0.4312	0.4447

The controls of all specifications include individual and meteorological characteristics. The individual characteristics include the hukou type, availability of pension, and health insurance. The meteorological characteristics include sunshine hours, relative humidity, wind speed, and precipitation. Robust standard errors clustered at the city level. Individual FE, City FE, Year FE, and Month FE denote the inclusion of individual fixed effects, city fixed effects, annual fixed effects, and monthly fixed effects, respectively. YES indicates that the corresponding fixed effect is controlled for, while NO indicates that it is not controlled for.

^**^P < 0.01, ^****^P < 0.01.

### 5.4 Excluding unpredictable shocks

In spring 2020, COVID-19 started to spread in China. The fear of contracting the virus and the uncertainty surrounding the pandemic have contributed to heightened levels of anxiety and stress. Moreover, the social isolation measures of epidemic prevention, including lockdowns, social distancing, and quarantine, have led to increased feelings of loneliness and isolation, contributing to depression. Therefore, we exclude 2020, the worst year of the epidemic. The results shown in column (2) of Table 5 indicate that even if the effects of the epidemic are excluded, air pollution still raises CES-D scores and has a negative impact on the mental health of middle-aged and older adults.

### 5.5 Comparing impacts across regions

Given the potential heterogeneity in PM2.5 concentration changes across different regions, we further stratify our sample into four major regions in China—Eastern, Central, Western, and Northeastern—to examine the impact of PM2.5 concentration on the mental health of middle-aged and older adults. However, due to the relatively small sample size in the Northeastern region (observations = 2,806), we focus our empirical analysis on the Eastern, Central, and Western regions. As depicted in Figures 1, 2, the Western regions experienced an increase in PM2.5 concentration from 2000 to 2020. In contrast, the Eastern region witnessed a significant decline in PM2.5 concentration over the same period. The results presented in Table 6 indicate that regions with more substantial pollution changes exhibit more pronounced negative impacts on mental health, thereby providing further evidence that air pollution is a significant contributing factor. Specifically, each unit increase in PM2.5 concentration is associated with a 0.0316-point increase in the CES-D score among middle-aged and older adults in the Western regions, respectively. Moreover, this differential impact across regions with varying pollution trends supports the argument that air pollution is a significant contributing factor to mental health outcomes.

**Table 6:** Comparing impacts across regions.

**Variables**	**Eastern region**	**Central region**	**Western region**
Pollution	−0.0405	0.0110	0.0316^***^
	(0.0394)	(0.0339)	(0.0064)
Constant	3.3836^***^	2.5590^***^	3.0620^***^
	(0.8354)	(0.7139)	(0.7447)
Controls	YES	YES	YES
Individual FE	YES	YES	YES
City FE	YES	YES	YES
Year FE	YES	YES	YES
Month FE	YES	YES	YES
Time trend	YES	YES	YES
Observations	10,926	13,331	12,959
Adj. R^2^	0.4062	0.6489	0.6616

The controls of all specifications include individual and meteorological characteristics. The individual characteristics include the hukou type, availability of pension, and health insurance. The meteorological characteristics include sunshine hours, relative humidity, wind speed, and precipitation. Robust standard errors clustered at the city level. Individual FE, City FE, Year FE, and Month FE denote the inclusion of individual fixed effects, city fixed effects, annual fixed effects, and monthly fixed effects, respectively. YES indicates that the corresponding fixed effect is controlled for, while NO indicates that it is not controlled for.

^****^P < 0.01.

### 5.6 Using alternative model specifications

Table 7 includes results for four more robustness checks using alternative model specifications. In column (1), we include year-month FE as a robust check. In column (2), we control for the city-month FE so that the estimated coefficients will be more accurate by accounting for potential omitted variable bias that varies across cities and over time. The results show that average air pollution affects mental health. Increases the CES-D score by 0.0347, indicating that a causal effect, large positive deviations from air pollution significantly deteriorate mental health. In column (3), we include the city-year FE as a robustness check. We aim to eliminate excessive variations in meteorological variables due to the data collection in the same city being usually conducted and focus on annual changes within each city. In column (4), we use the significance level of the estimates from the two-way clustering standard by city and interview month.

**Table 7:** A series of robustness checks: using alternative model specifications.

**Variables**	**(1)**	**(2)**	**(3)**	**(4)**
Pollution	0.0310^***^	0.0347^***^	0.0185^**^	0.0022^*^
	(0.0071)	(0.0073)	(0.0062)	(0.0010)
Constant	3.0093^***^	2.5349^***^	2.7029^***^	0.8490^***^
	(0.3463)	(0.3877)	(0.5587)	(0.2831)
Controls	YES	YES	YES	YES
Individual FE	YES	YES	YES	YES
City FE	YES	NO	NO	YES
Year FE	NO	NO	NO	NO
Month FE	NO	NO	NO	NO
Time trend	YES	YES	YES	YES
City-by-year FE	NO	NO	YES	NO
City-by-month FE	NO	YES	NO	NO
Year-by-month FE	YES	YES	YES	NO
Observation	40,791	40,750	40,771	40,792
Adj. R^2^	0.4319	0.4362	0.4431	0.4136

The controls of all specifications include individual and meteorological characteristics. The individual characteristics include the hukou type, availability of pension, and health insurance. The meteorological characteristics include sunshine hours, relative humidity, wind speed, and precipitation. Robust standard errors clustered at the city level. Individual FE, City FE, Year FE, and Month FE denote the inclusion of individual fixed effects, city fixed effects, annual fixed effects, and monthly fixed effects, respectively. YES indicates that the corresponding fixed effect is controlled for, while NO indicates that it is not controlled for.

^*^P < 0.1, ^**^P < 0.05, ^****^P < 0.01.

## 6 Heterogeneity analyses

To identify populations whose mental health is more vulnerable to air pollution, we investigate the heterogeneous effect of air pollution on depressive disorder among sample populations with different innate characteristics and acquired differences.

### 6.1 Heterogeneity by age

Some relevant literature pointed out that air pollution exposure may lead to a higher risk of depression later in life (50). We further divided the respondents into three groups: middle-aged (ages 45–60), older adults (ages 61–70), and old seniors (ages 71 and above), and conducted regression analyses for each group. Results in Table 8 indicate the heterogeneous effect of air pollution on mental health among different age groups. We find that middle-aged and older seniors are more susceptible to the adverse effects of air pollution on their mental health. Furthermore, the marginal impact on the mental health of older seniors is slightly higher than that on middle-aged individuals. However, for individuals aged between 61 and 70, referred to as the older adults, the air pollution does not have significant effects on the CES-D scores. For middle-aged individuals, frequent social activities, commuting, and outdoor work increase their exposure to polluted environments, thus rendering them more susceptible to the adverse effects of air pollution. In contrast, older seniors are more likely to rely on family and social care, with lower levels of autonomy in daily living and weaker adaptive capacity to environmental changes, making them more vulnerable to the impacts of air pollution. Consequently, social and family support play more critical roles in the mental health of this age group.

**Table 8:** Heterogeneous by age.

**Variables**	**45–60**	**61–70**	**>71**
Pollution	0.0288^**^	0.0113	0.0312^***^
	(0.0113)	(0.0129)	(0.0069)
Constant	3.0287^***^	2.5042^***^	3.4825^***^
	(0.0113)	(0.5107)	(0.7355)
Controls	YES	YES	YES
Individual FE	YES	YES	YES
City FE	YES	YES	YES
Year FE	YES	YES	YES
Month FE	YES	YES	YES
Time trend	YES	YES	YES
Observations	19,027	10,914	5,284
Adj. R^2^	0.4341	0.6773	0.6549

The controls of all specifications include individual and meteorological characteristics. The individual characteristics include the hukou type, availability of pension, and health insurance. The meteorological characteristics include sunshine hours, relative humidity, wind speed, and precipitation. Robust standard errors clustered at the city level. Individual FE, City FE, Year FE, and Month FE denote the inclusion of individual fixed effects, city fixed effects, annual fixed effects, and monthly fixed effects, respectively. YES indicates that the corresponding fixed effect is controlled for, while NO indicates that it is not controlled for.

^**^P < 0.05, ^****^P < 0.01.

### 6.2 Heterogeneity by gender

Some studies suggest that the impact of air pollution on mental health has a significant modification by gender (51). Therefore, we proceed to examine the heterogeneous impact of air pollution on mental health between males and females. Results in column (1) of Table 9 show that males are more prone to mental illness induced by air pollution than females. The observed gender disparity may stem from several interrelated factors. Behaviorally, due to occupational differences, men are often more exposed to polluted environments. Furthermore, psychological literature suggests that men are less likely to seek emotional support, potentially exacerbating pollutant-induced stress effects.

**Table 9:** Heterogeneity analyses by gender, marital status (widowed or not), and education level.

**Variables**	**Gender**	**Widowed or not**	**Education**
	**(male = 1)**	**(widowed = 1)**	**(low level = 1)**
Pollution^*^Dummy	0.0183^**^	0.0306^***^	0.0093
	(0.0079)	(0.0061)	(0.0071)
Pollution	0.0227^**^	0.0312^***^	0.0241^**^
	(0.0093)	(0.0070)	(0.0099)
Dummy	−0.1925	0.6881	0.0135
	(0.1787)	(0.1341)	(0.3962)
Constant	3.0699^***^	2.9798^***^	2.9672^***^
	(0.3573)	(0.3507)	(0.3506)
Controls	YES	YES	YES
Individual FE	YES	YES	YES
City FE	YES	YES	YES
Year FE	YES	YES	YES
Month FE	YES	YES	YES
Time trend	YES	YES	YES
Observations	40,791	40,791	40,791
Adj. R^2^	0.4319	0.4319	0.4319

The controls of all specifications include individual and meteorological characteristics. The individual characteristics include the hukou type, availability of pension, and health insurance. The meteorological characteristics include sunshine hours, relative humidity, wind speed, and precipitation. Robust standard errors clustered at the city level. Individual FE, City FE, Year FE, and Month FE denote the inclusion of individual fixed effects, city fixed effects, annual fixed effects, and monthly fixed effects, respectively. YES indicates that the corresponding fixed effect is controlled for, while NO indicates that it is not controlled for.

^**^P < 0.05, ^****^P < 0.01.

### 6.3 Widowed or not

The psychological state of widowed older adults can indeed be more fragile compared to their married or non-widowed counterparts (52). This fragility may stem from the significant emotional loss and the subsequent adjustment to life without their spouse, which can profoundly impact their mental health. Results in column (2) of Table 9 show that the widowed older adults are more susceptible to the effects of air pollution on mental health.

### 6.4 Heterogeneity by education level

Education may equip individuals with more knowledge on how to cope with air pollution (53). The survey population in CHARLS was mostly born in the 1970s, when educational attainment was generally low. Based on this, we next define a dummy variable of low educational attainment based on the 9-year compulsory education in the interviewers. Respondents who have obtained at least 9 years of education are classified into the higher education group, and those with < 9 years of education are classified into the lower education group. Results are displayed in column (3) of Table 9. We find that educational level does not significantly moderate the impact of air pollution on the mental health of middle-aged and older adults. Existing evidence suggests that air pollution impacts mental health primarily through biological mechanisms, including neuroinflammation, oxidative stress, and hypothalamic–pituitary–adrenal (HPA) axis dysregulation, that are independent of conscious awareness or behavioral mediation (54). Furthermore, physiological vulnerabilities associated with aging, such as increased prevalence of chronic diseases, may reduce the differential protective effects of education across exposure groups. Notably, individuals with higher education levels may exhibit greater environmental risk perception and awareness, potentially increasing psychological stress or anxiety related to pollution exposure (55). Consequently, educational attainment may not significantly moderate the relationship between air pollution exposure and mental health outcomes in the older adults population.

## 7 Underlying mechanisms

### 7.1 Sleep quality

Existing literature has suggested that air pollution exposure impairs physical health, which can lead to deteriorating mental health (56). Specifically, air pollution exposure may also change an individual's lifestyle and health behaviors. Studies have shown that exposure to higher concentrations of particulate matter (PM2.5) is associated with poor sleep quality (57). Notably, poor sleep quality is associated with an increased risk of mental health issues. Poor and insufficient sleep increases negative emotional responses to stressors, decreases positive emotions, and may exacerbate psychiatric symptoms (58).

Scientific studies recommend that older adults, typically those aged 65 and older, should aim for 7–8 h of sleep per night (59). However, we take individual variations into account. Specifically, some older adults may feel natural and energized waking up after 6 h of sleep, while others may need up to 9 h of sleep to feel rejuvenated. Moreover, CHARLS collects information on the duration of sleep at night over the past month. Based on this information, we construct a sleep quality indicator (sleep) based on their sleep duration, i.e., if a respondent sleeps more than 6 h and < 9 h during the night, we consider that he/she has good sleep quality, which takes the value of 1. Otherwise, it is equal to zero. Further, we divide the sample into two groups: the middle-aged group (45–65) and the older adult group (>65). We next estimated whether air pollution affects the status of individual health behaviors and sleep quality using a linear probability model. Results are displayed in column (1) and column (2) of Table 10. The air pollution exposure has a significant negative impact on older adults, but not on the middle-aged group (45–65 years old), suggesting that severe air pollution impairs sleep quality and affects the lifestyles of older adults. Poor sleep quality, in turn, can negatively impact mental health, leading to increased anxiety and depression.

**Table 10:** Mechanisms underlying the depressive effects of PM2.5: sleep quality and cardiopulmonary status.

**Variables**	**Sleep quality (40–65)**	**Sleep quality (>65)**	**Cardiopulmonary status (45–65)**	**Cardiopulmonary status (>65)**
Pollution	0.0032	−0.007^***^	0.0021	−0.0064^*^
	(0.0026)	(0.0025)	(0.0028)	(0.0034)
Constant	0.2791^*^	0.2960	0.8837^***^	0.7797^***^
	(0.1491)	(0.2296)	(0.1361)	(0.1927)
Controls	YES	YES	YES	YES
Individual FE	YES	YES	YES	YES
City FE	YES	YES	YES	YES
Year FE	YES	YES	YES	YES
Month FE	YES	YES	YES	YES
Time trend	YES	YES	YES	YES
Observations	27,139	10,458	27,139	10,458
Adj. R^2^	0.3296	0.3844	0.2450	0.2668

The controls of all specifications include individual and meteorological characteristics. The individual characteristics include the hukou type, availability of pension, and health insurance. The meteorological characteristics include sunshine hours, relative humidity, wind speed, and precipitation. Robust standard errors clustered at the city level. Individual FE, City FE, Year FE, and Month FE denote the inclusion of individual fixed effects, city fixed effects, annual fixed effects, and monthly fixed effects, respectively. YES indicates that the corresponding fixed effect is controlled for, while NO indicates that it is not controlled for.

^*^P < 0.1, ^****^P < 0.01.

### 7.2 Individual cardiopulmonary health status

Existing literature suggests that air pollution impairs physical health, which can lead to deteriorating mental health (60). We estimate whether air pollution affects the cardiopulmonary health status of individuals. Based on this, CHARLS asks each interviewee whether they feel chest pain when climbing stairs (uphill) or walking quickly, which is usually used to assess cardiopulmonary health conditions. We define a dummy variable of individual cardiopulmonary health status that he/she feels well in the chest and walks quickly, which takes the value of 1. Otherwise, it is equal to zero. We estimate the effects of air pollution on this dummy variable using a linear probability model and differentiate the effects between the middle-aged and older adults. The primary findings are presented in columns (3) and (4) of Table 10. Specifically, a one-unit increase in PM2.5 concentration is associated with a 0.0064 rise in the probability of cardiovascular and cerebrovascular deterioration among the older adult population. This suggests that even minor fluctuations in PM2.5 levels can exert a significant impact on the cardiopulmonary health of this vulnerable group. For older adults who already suffer from cardiovascular and cerebrovascular diseases, the heightened risk may lead to a higher likelihood of disease exacerbation. This, in turn, indicates that air pollution not only directly harms the cardiopulmonary health of older adults but also indirectly exacerbates their mental health status through the worsening of physical health conditions. However, the effect of air pollution on the cardiopulmonary health status of the middle-aged group is not significant.

### 7.3 Life satisfaction

Air pollution has been found to impact life satisfaction negatively (61). A study explored the effect of ambient air pollution on individual levels of subjective wellbeing. This suggests that even relatively low levels of PM10 air pollution can adversely affect people's subjective assessments of well-being and its effects on physical health.

Based on this, we estimate the implication of air pollution on self-reported subjective wellbeing. The CHARLS asked each respondent about their recent life satisfaction. The self-reported future confidence is a discrete numerical variable that takes integer values from 1 to 5. Scoring values are as follows: completely satisfied, very satisfied, somewhat satisfied, not at all satisfied. The mean reported life satisfaction in the survey is 2.82 out of 5. The results are in columns (1) and (2) of Table 11. A one standard deviation rise in PM2.5 concentration results in a decrease of 0.034 units in life satisfaction, which means a decline in an individual's overall sense of wellbeing and contentment with life. Hence, it is crucial to improve air quality to enhance the quality of life for older adults. High PM2.5 concentrations can render the air turbid and reduce visibility, thereby restricting outdoor activities for older adults. This diminishes their comfort and convenience in daily life.

**Table 11:** Mechanisms underlying the depressive effects of PM2.5: life satisfaction.

**Variables**	**Life satisfaction (40–65)**	**Life satisfaction (>65)**
Pollution	−0.0025	−0.0339^***^
	(0.0103)	(0.0131)
Constant	2.7621^***^	2.6937^***^
	(0.3272)	(0.5192)
Controls	YES	YES
Individual FE	YES	YES
City FE	YES	YES
Year FE	YES	YES
Month FE	YES	YES
Time trend	YES	YES
Observations	11,604	4,932
Adj. R^2^	0.2950	0.1883

The controls of all specifications include individual and meteorological characteristics. The individual characteristics include the hukou type, availability of pension, and health insurance. The meteorological characteristics include sunshine hours, relative humidity, wind speed, and precipitation. Robust standard errors clustered at the city level. Individual FE, City FE, Year FE, and Month FE denote the inclusion of individual fixed effects, city fixed effects, annual fixed effects, and monthly fixed effects, respectively. YES indicates that the corresponding fixed effect is controlled for, while NO indicates that it is not controlled for.

^****^P < 0.01.

### 7.4 Cognitive competence

Cognitive ability predicts health outcomes, academic achievement, and mental health. Therefore, we try to figure out the effect of air pollution exposure on human cognition. This has significant implications for multiple areas of policy. The scope of cognitive evaluation includes date recognition ability, numeracy, and situational memory capacity (62). Specifically, in the assessment of date recognition, respondents were asked to answer the current day of the week, date, month, season, and year, with each correct answer earning 1 point, for a total of five points. In the numeracy assessment, respondents were asked to answer 100 minus 7, with five consecutive subtractions, where 1 point was awarded for each correct answer, totalling 5 points. In the assessment of drawing ability, the respondent draws the picture that they have seen and scores 1 point for a successful drawing. The assessment has a total score of 11, with higher scores representing greater cognitive ability. The results are displayed in columns (1) and (2) of Table 12. A one standard deviation increase in PM2.5 concentration is associated with a decrease in cognitive ability scores by 3.0361 for middle-aged individuals yet exhibits no significant impact on older adults. We found that air pollution impairs cognitive performance in both middle-aged and older age groups, but to a greater extent in older adults than in middle-aged adults.

**Table 12:** Mechanisms underlying the depressive effects of PM2.5: cognitive competence.

**Variables**	**Cognitive competence (45–65)**	**Cognitive competence (>65)**	**Cognitive Index (45–65)**	**Cognitive Index (>65)**
Pollution	−3.0361^**^	−2.0299	−0.0978	−0.2610^***^
	(1.9625)	(1.4990)	(0.8135)	(1.3204)
Constant	50.6582^***^	239.0744^***^	217.0952^***^	331.2852^***^
	(71.6456)	(162.6932)	(52.17548)	(84.5498)
Controls	YES	YES	YES	YES
Individual FE	YES	YES	YES	YES
City FE	YES	YES	YES	YES
Year FE	YES	YES	YES	YES
Month FE	YES	YES	YES	YES
Time trend	YES	YES	YES	YES
Observations	27,139	19,408	26,376	10,458
Adj. R^2^	0.1108	0.1389	0.5084	0.5013

The controls of all specifications include individual and meteorological characteristics. The individual characteristics include the hukou type, availability of pension, and health insurance. The meteorological characteristics include sunshine hours, relative humidity, wind speed, and precipitation. Robust standard errors clustered at the city level. Individual FE, City FE, Year FE, and Month FE denote the inclusion of individual fixed effects, city fixed effects, annual fixed effects, and monthly fixed effects, respectively. YES indicates that the corresponding fixed effect is controlled for, while NO indicates that it is not controlled for.

^**^P < 0.05, ^****^P < 0.01.

Further, we construct an indicator of cognitive ability in the following Equation 3. CogIndex is the proportion of the overall number of indicators in which an individual has a health risk for each of the given health indicator ratings (63),


(3) $$\begin{array}{l} CogIndex_{i}=\frac{\sum_{j=1}^{N_{j}}D_{ij}}{N} \end{array}$$

Where *CogIndex*_*i*_ is the cognitive ill-health index. *N* is the total score of the cognitive health index variable (in our research, *N* = 11). *D*_*ij*_ is the value corresponding to the jth variable reflecting the cognitive health ability of an individual i. *D*_*ij*_ = 1 means the jth cognitive ability variable is healthy. *D*_*ij*_ = 0 means that the jth cognitive ability variable is in a state of health risk. Therefore, the larger value of *CogIndex*_*i*_ indicates that the individual's cognitive ability is more excellent. The results are presented in columns (3) and (4) of Table 12. A one standard deviation increases in PM2.5 concentration is associated with a 0.3307 reduction in the cognitive index for older adults. Our findings suggest that exposure to air pollution is correlated with cognitive decline, which is further associated with an elevated risk of depression, particularly for older adults. Meanwhile, relevant studies have indicated that individuals exhibiting higher levels of depressive symptoms at the initial stage tend to experience a more pronounced cognitive decline over time, suggesting a bidirectional relationship (64, 65). This finding underscores the complexity of the interplay between air pollution, cognitive function, and mental health. The bidirectional relationship between cognitive decline and depression has important implications for understanding the impact of air pollution on mental health. While our study primarily focused on cognitive decline as a mechanism linking air pollution to depression, the reverse pathway from depression to cognitive decline cannot be ignored.

## 8 Further analyses: How to minimize health risks from air pollution

### 8.1 Private avoidance behaviors

The high internet and mobile phone penetration in China provides a great opportunity to investigate pollution information using digital sources. The network significantly expands access to pollution information and elevates people's awareness about pollution issues. This information sharing may influence the avoidance behaviors of individuals who can take action. Like Google Trends, the Baidu Index is a data analysis tool provided by Baidu, the most widely used search engine in China. It allows users to see the research frequency of specific keywords in each city over a specified period since 2011. We focus on the search index for “haze pollution,” the internet catchphrase for air pollution, to illustrate personal access to the pollution information.

We estimate the moderating effects of private avoidance behaviors on the impact of air pollution on mental health. The results are shown in columns (1) and (2) of Table 12. The wider personal access to pollution-related information, the more effective they can be in counteracting the damage to mental health caused by air pollution, both for middle-aged and older adults. The results were not statistically significant for older adults. We assume that it may be related to the older population's lower proficiency in Internet use.

### 8.2 The filtering effect of urban vegetation density

Vegetation, especially trees and shrubs, can act as a natural air filter. They capture particulate matter on their leaves and surfaces, which helps to reduce the concentration of these particles in the air. Hence, we estimate the filtering effect of urban vegetation density (we call the urban forest).^5^ We use the NDVI indicator (normalized difference vegetation index), which is a graphical indicator used to analyze remote sensing measures of vegetation density to measure whether a city is an urban forest city and investigate the modifying role of greenness.

The formula for NDVI is:


(4) $$\begin{array}{l} NDVI=\frac{NIR-RED}{NIR+RED} \end{array}$$

Where NIR is the near-infrared light reflected by vegetation, and RED is the visible light reflected. NDVI values range from −1 to +1. Healthy vegetation can reflect a lot of near-infrared light (NIR) and absorb most infrared light (RED). Based on this, a high value (closer to +1) indicates high green, healthy vegetation concentrations, while lower values (closer to −1) indicate sparse or unhealthy vegetation or non-vegetative surfaces.

The NDVI data are obtained from the NASA Moderate Resolution Imaging Spectroradiometer (MODIS) product MOD13Q1.061, which provides vegetation indices at a spatial resolution of 1 km. We obtained monthly data based on when and where respondents were interviewed. We further matched the data with the NDVI values of the respondents' locations at the time of the survey. It is generally accepted that an NDVI value above 0.6 indicates that the location is rich in vegetation and appears very green.^6^ Further, we defined a dummy variable for urban forest cities, where a value of 1 indicates that the NDVI value in the area is above 0.6 (i.e., the area has dense vegetation), and a value of 0 indicates otherwise. We examined the interactions of this variable with urban air quality variables. The results are displayed in columns (3) and (4) of Table 13; compared with those interviewees in non-urban forest cities, respondents are less mentally impaired by air pollution. The improvement of mental health for older adults is more pronounced than for the middle-aged. The evidence points out that greenness, as a nature-based intervention, can mitigate the residential air pollution of physiological stress.

**Table 13:** The moderating effects of air pollution exposure: private avoidance behaviors and urban vegetation density.

**Variables**	**(1)**	**(2)**	**(3)**	**(4)**
	**(45–65)**	**(>65)**	**(45–65)**	**(>65)**
Pollution^*^Baidu	−0.01361^**^	0.0014		
	(0.0059)	(0.0024)		
Baidu	0.0367^*^	−0.0213		
	(0.0207)	(0.0257)		
Pollution	0.1242^***^	0.0545	0.0145^***^	0.0337^***^
	(0.0342)	(0.0309)	(0.0050)	(0.0028)
Pollution^*^NDVI			−0.0219^**^	−0.0390^**^
			(0.0106)	(0.0195)
NDVI			−0.5661^**^	−0.0334^*^
			(0.2686)	(0.0186)
Constant	2.1083^***^	2.3639^***^	3.5508^***^	3.2983^***^
	(0.6332)	(1.1396)	(0.4934)	(0.6626)
Controls	YES	YES	YES	YES
Individual FE	YES	YES	YES	YES
City FE	YES	YES	YES	YES
Year FE	YES	YES	YES	YES
Month FE	YES	YES	YES	YES
Time trend	YES	YES	YES	YES
Observations	9,549	3,682	27,139	10,458
Adj. R^2^	0.4393	0.3757	0.4396	0.4019

NDVI stands for Normalized Difference Vegetation Index. The controls of all specifications include individual and meteorological characteristics. The individual characteristics include the hukou type, availability of pension, and health insurance. The meteorological characteristics include sunshine hours, relative humidity, wind speed, and precipitation. Robust standard errors clustered at the city level. Individual FE, City FE, Year FE, and Month FE denote the inclusion of individual fixed effects, city fixed effects, annual fixed effects, and monthly fixed effects, respectively. YES indicates that the corresponding fixed effect is controlled for, while NO indicates that it is not controlled for.

^*^P < 0.1, ^**^P < 0.05, ^****^P < 0.01.

**Figure 6:**
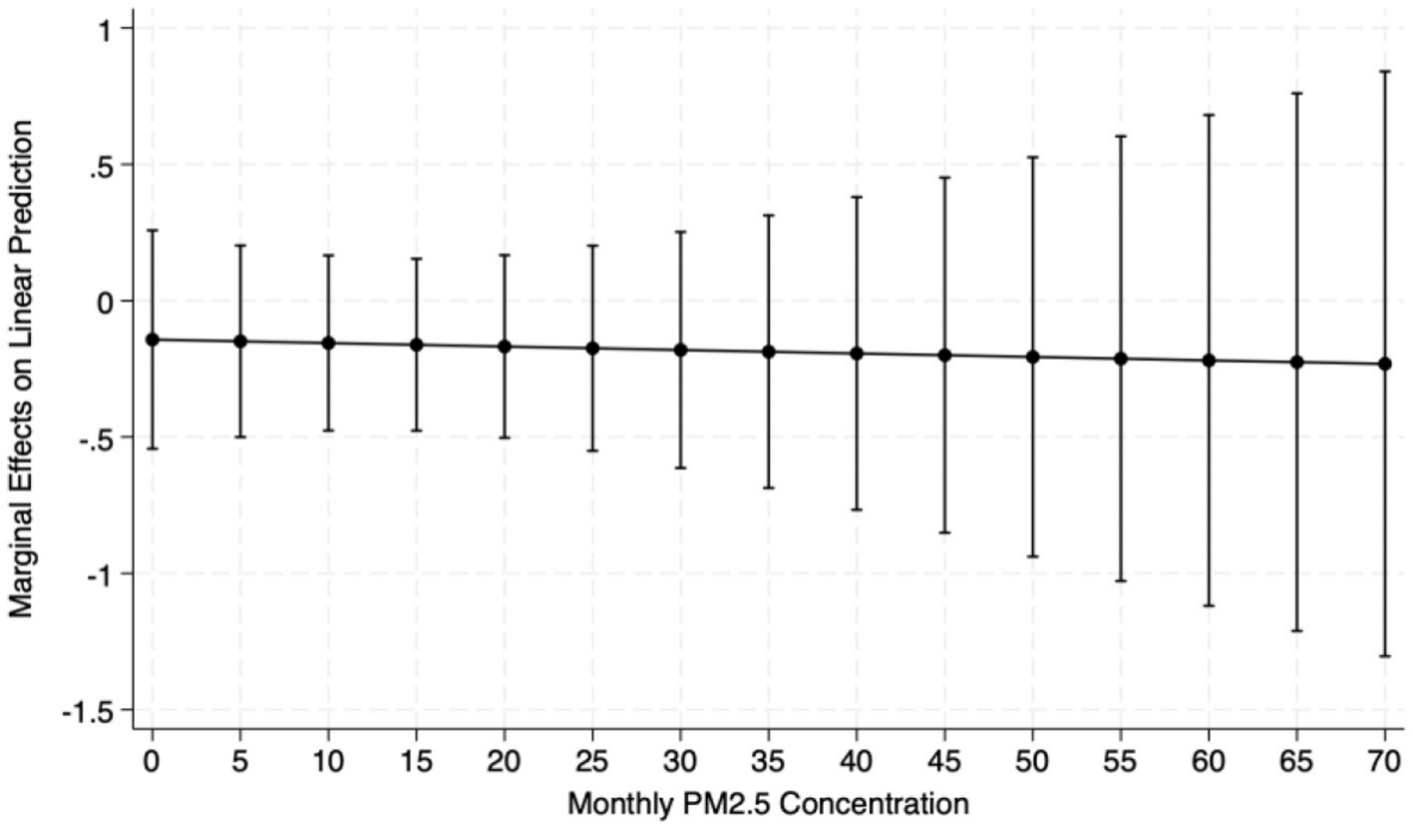
Marginal effects of NDVI on CES-D. The NDVI stands for Normalized Difference Vegetation Index. This figure presents the marginal effects of the interaction term (NDVI*Pollution). The influence of high vegetation density on the marginal impact of PM2.5 on the CES-D scores of middle-aged and older adults persists as PM2.5 concentrations increase.

Furthermore, we have conducted additional calculations to explore the marginal effect of high vegetation density (i.e., NDVI = 1) on the CES-D scores of middle-aged and older adults under varying concentrations of PM2.5. As illustrated in Figure 6, which presents the marginal effects of the interaction term, the influence of high vegetation density on the marginal effect of PM2.5 on the CES-D scores of middle-aged and older adults persists as PM2.5 concentrations increase. This effect significantly mitigates the CES-D scores, thereby improving psychological health status, although the magnitude of change is not substantial. These findings further verify the notion that a higher degree of greening can effectively ameliorate the impact of air pollution on the mental health of the middle-aged and older adults.

## 9 Conclusions

Nearly 14% of the global burden of disease has been attributed to chronic depression and other common mental disorders (66). Mental disorders have become a severe threat to public health around the globe. In our paper, we explore the impacts of air pollution on mental health among middle-aged and older adults by applying the high-quality Chinese middle-aged and older adults survey database. We ensure the identification of the external variation in air pollution within an individual after meteorological characteristics, individual characteristics, a series of fixed effects, and common time trends.

The heterogeneous analyses on the mental health effects of air pollution help to identify more vulnerable populations. Females are more vulnerable than males to the effects of air pollution on their mental health conditions. Understanding these gender differences is crucial for developing targeted interventions and policies to protect and improve mental health in the face of increasing air pollution worldwide. Both middle-aged and older adults are susceptible to air pollution. However, the effect of air pollution on the oldest-old is not significant, possibly due to limited mobility and less time for outdoor activities. Widowed individuals are affected more than married ones, which complements the existing literature that air pollution imposes a far-reaching influence on mentally relevant costs for the widowed. The compounded stress of widowhood and exposure to air pollution can exacerbate mental health issues in this population.

Regarding the underlying mechanisms, we consider the differences in somatic functions and differentiate between the middle-aged and older adults. Air pollution is found to significantly reduce the length of sleep at night and life satisfaction among older adults. The cardiopulmonary health status of the aged, likely affected by air pollution, becomes worse. Air pollution significantly reduces cognitive performance in middle-aged and older adults, which is also corroborated by some literature (67). Furthermore, we continue to analyze people's avoidance behaviors for air pollution, including vegetation shielding and information retrieval. We found that the lusher vegetation in urban areas, which usually means a higher NDVI value, can effectively filter out part of the air pollutants and increase psychological wellbeing. Expanding access to information on air pollution can also help people take timely measures to avoid air pollution. Policymakers should prioritize increasing tree coverage in polluted districts, as vegetation can act as a natural barrier to filter out air pollutants and enhance psychological wellbeing. Mandating green buffer zones around older adults communities could provide a protective environment for this vulnerable population. Additionally, enhancing targeted mental health interventions for women and widowed persons is essential to address the disproportionate impacts of air pollution on their mental health. Expanding access to air pollution information and promoting public awareness campaigns can empower individuals to take timely measures to avoid exposure.

Exploring the impact of air pollution on mental health deepens our understanding of the socioeconomic consequences of climate change. While our study is based on a Chinese dataset, the findings have broader implications for other aging societies worldwide. The increasing prevalence of mental health issues among middle-aged and older adults, coupled with the growing threat of air pollution, is a global concern. Our results highlight the importance of integrating mental health considerations into air pollution control measures, such as emission reductions, the implementation of green infrastructure, and strategic urban planning. Additionally, evaluating the influence of public health policies and regulations on diminishing air pollution levels and enhancing overall population health outcomes is critical. We consider that there are important issues for future research. Furthermore, the geographic distribution of the survey participants in our study may not align perfectly with regions experiencing substantial increases in PM2.5 levels. This aspect could influence the spatial applicability of our results. Future studies could further enhance the external validity by ensuring a participant sample that more closely mirrors the demographics of areas with significant PM2.5 exposure changes.

## Data Availability

The original contributions presented in the study are included in the article/supplementary material, further inquiries can be directed to the corresponding author.

## Footnotes

^1^The website is https://www.cnemc.cn.

^2^The website is: https://sites.wustl.edu/acag/datasets/surface-pm2-5/.

^3^Given the serious lack of income data, we use the possession of a pension as a measure of financial security for the middle-aged and older adults. If a person has one or more of these types of pensions, we consider him/ her to have pension. They include pension for public servants, public institution employees, and basic pension for enterprise employees; supplementary pension insurance(annuity); urban and rural resident pension, new rural resident pension and urban resident pension; pension for land-expropriated farmers; life insurance; commercial pension insurance and other pension.

^4^If a person has one or more of these nine types of medical insurance, we consider him/her to have medical insurance. They include urban employee medical insurance; urban resident medical insurance; new cooperative medical insurance (it's also called he-zuo-yi-liao); urban and rural resident medical insurance; government medical insurance (it's also called gong-fei); medical aid; private medical insurance: purchased by work unit; Private medical insurance: purchased by individual; urban non-employed person's health insurance; other medical insurance(specify).

^5^The urban forests refer to the collection of trees and other vegetation with urban areas, which are valuable components of the urban environment.

^6^ https://climatedataguide.ucar.edu/climate-data/ndvi-normalized-difference-vegetation-index-noaa-avhrr
